# 1933. Assessment of Effectiveness of mRNA COVID-19 Vaccines Among Health Care Personnel: Monroe County, NY December 2020-March 2022

**DOI:** 10.1093/ofid/ofac492.1560

**Published:** 2022-12-15

**Authors:** Savanah Russ, Christopher J Myers, Infectious Diseases, Erin Licherdell, Thomas Peer, Christine Hurley, Cathleen Concannon, Acacia Bowden, Ellen Chinchilli, Alan Alvarado, Ghinwa Dumyati

**Affiliations:** Center for Community Health & Prevention, University of Rochester Medical Center, Rochester, New York; University of Rochester, Rochester, New York; University of Rochester Medical Center, Rochester, New York; Center for Community Health & Prevention, University of Rochester Medical Center, Rochester, New York; University of Rochester, Center for Community Health and Prevention, Rochester, New York; University of Rochester Medical Center, Rochester, New York; University of Rochester School of Medicine and Dentistry, Rochester, New York; University of Rochester School of Medicine and Dentistry, Rochester, New York; Center for Community Health & Prevention, University of Rochester Medical Center, Rochester, New York; University of Rochester Medical Center, Rochester, New York

## Abstract

**Background:**

Risk of infection with SARS-CoV-2 has remained high among health care personnel (HCP) throughout the pandemic, due to both exposure in the community and occupational settings. While vaccine uptake among health care workers is high, real-world continual monitoring of vaccine effectiveness (VE) among this population is crucial for informing future vaccination and prevention efforts.

**Methods:**

Data for this analysis came from a test-negative case-control study conducted among HCP working at two acute care hospitals in Monroe County, NY from December 2020 through March 2022, performed as part of the CDC Emerging Infections Program. Case participants were identified as HCP who had at least one COVID-19 like symptom, and a positive polymerase-chain-reaction (PCR) SARS-CoV-2 test during the study time period. Control participants had a negative SARS-CoV-2 PCR test, regardless of presence of COVID-19 like symptoms. Cases and controls were matched based on the study week of their test date. Conditional logistic regression was used to assess vaccine effectiveness against symptomatic infection. Effectiveness was assessed between December 2020-May 2021, May 2021-October 2021, and October 2021-March 2022.

**Results:**

From December 28^th^, 2020 through March 12^th^, 2022, 881 cases and 1794 controls were enrolled. Vaccine effectiveness against symptomatic infection was greatest from December 2020 through May 2021, with mRNA complete series effectiveness at 93.1% (95% CI: 86.9%-96.3%) with complete series VE falling to 25.1% (95% CI: 0.0%-50.9%) during May 2021-October 2021. Waning immunity following receipt of second dose was observed across all time periods. Vaccine effectiveness following receipt of one booster vaccine was found to be 59.2% (95% CI: 43.5-70.6), with evidence of waning immunity two months from receipt of the booster (VE: 46.6%; 95% CI: 14.6%-66.7%).

**Conclusion:**

Protection provided by the COVID-19 mRNA vaccines against symptomatic infection is highly variable among HCP, based on the circulating dominant variant and the time since receipt of each dose. Monitoring of vaccine effectiveness, as well as waning immunity, among this high-risk population is essential to guide future vaccine policies.

**Disclosures:**

**christopher J. Myers, MS, Infectious diseases**, Pfizer: Grant/Research Support **Ghinwa Dumyati, MD**, Pfizer: Grant/Research Support.

